# The Events of a Critical Period

**Published:** 1921-04-16

**Authors:** 


### KING EDWARD'S HOSPITAL FUND FOR LONDON.

### The Events of a Critical Period.

The annual meeting of the President and General Council of King Edward's Hospital Fund for London, to receive the accounts and the report for the year 1920, was held at St. James's Palace on April 12, His Royal Highness the Prince of Wales being in the chair.

Lord Revelstoke (Honorary Treasurer), in presenting the accounts of receipts and expenditure and the balance-sheet for the year ending December 31, 1920, said that a full statement as to the general receipts was made at the meeting in December last, and to the particulars then given there is but little to add. If, following the usual practice, general legacies be included in the total "General Receipts," but not the amount received from the Lewis Estate and carried specially to capital, the total "General Receipts" for the year ended December 31 last enabled the Fund to provide nearly £193,000 out of the ordinary distribution of £200,000—a very gratifying result when it is borne in mind that this distribution exceeds by £42,000 the amount distributed in the year immediately preceding the war.

The emergency distribution of £250,000 made in July 1920 was provided from the reserve funds of the corporation. The credit side of the accounts shows that £256,935 in all was drawn from the reserve in 1920, the extra £6,935 being the sum required to complete the amount of the ordinary distribution. The necessary realisation of securities entailed a loss of £8,627, or about $4\frac{1}{3}$ per cent. of the book cost. The effect of this distribution has been to reduce the available funds by about one-fourth, with a consequent loss in income of about £16,000 per annum. This emergency distribution was a special measure of an urgent nature, imposed on the Fund by the necessity of affording time in which it might be possible to arrange for the reorganisation of hospital finances in general. The Finance Committee feel that such a step cannot be repeated without serious detriment both to the Fund and, consequently, to the best interests of the hospitals. Lord Revelstoke said a great step towards a permanent solution of the problem of the voluntary hospitals had been taken by the appointment of Lord Cave's Committee, and the hopes of its success in what must of necessity be a very difficult task have been strengthened by the appearance of the very practical proposals contained in the committee's interim report. It is particularly encouraging that the evidence taken by the committee has tended to convince them of the importance of every effort being made to continue and to preserve the voluntary system.

### Special Features of the Year.

The Chairman of the Executive Committee (Lord Stuart of Wortley) moved the adoption of the report of the Council for the year 1920. He said that the report was a long one, but neither controversial nor, he thought, otherwise disputable; for it was in the main historical and narrative. As such, it recounts the events of a most critical period in the history of the Fund. It recalls the exceptional events of the year: the absence of the President from the country for nearly two-thirds of the year on a mission which had become part of the history of the Empire; the crisis in the finances of the hospitals of the London area; the great contribution of £250,000 by the British Red Cross and the Order of St. John; the Fund's own emergency distribution of £250,000 from reserve; the consequent total allocation, including the ordinary distribution of £200,000, of no less than £700,000 in the year; the adoption of the policy of advocating such measures of relief only as should least tend to supersede or impair the voluntary system of hospital management; the appointment of Lord Cave's Committee; and all the consequent activities, still going on.

#### Receipts, Distribution, and Expenses.

The summary of the receipts and distribution shows that the total receipts for the year were £480,431, of which £255,365 was received from the Joint Committee of the British Red Cross and Order of St. John, and accrued interest on unpaid grants. £9,229 was from contributions to capital and £215,838 from general receipts. The amount distributed was £700,000, made up as follows: Surplus Red Cross funds, £250,000; emergency distribution, £250,000; ordinary distribution, £200,000. [Details of these grants were given in The Hospital of December 18 and 25.] The report calls attention to the fact that the Distribution Committee, in forwarding the emergency grants last July, urged on the hospitals the need for considering methods of improving their finances, including that of payment by patients, a method which has been widely adopted by the hospitals, both before and after that date, and has brought in a considerable addition to their income during 1920. The problem of hospital finance was continuously before the Executive Committee throughout the year, and the Hon. Secretaries had interviews with the British Hospitals Association, the Ministry of Health, and the National Relief Fund, and joined the Joint Committee, the League of Mercy, and the Hospital Sunday and Saturday Funds in considering proposals to increase the permanent income of the hospitals. The Council expresses appreciation of the National Relief Fund distribution to hospitals, £200,000 of which was allocated to London hospitals. These grants and the King's Fund emergency distribution have helped the hospitals to tide over immediate difficulties.

The total sum distributed by the Fund during the last ten years is £2,299,500; and since the foundation of the Fund, £3,588,416. The average annual distribution during the whole period of twenty-four years has been £149,517 17s. During 1920 the amount spent on the administration of the Fund, including the Red Cross distribution, was £5,484 9s. 4d., or £1 2s. 10d. per £100 of the total received, as compared with £4,085 17s. 9d., or £2 0s. 6d. per £100 in the previous year. The average yearly outlay in this respect for the whole period since the inauguration of the Fund has been £3,059, the corresponding percentage of the receipts being £1 5s. 3d., or less than $3\frac{1}{4}$d. in every £1 received.

#### Charitable Contributions and Income-tax.

In view of the allowance made for charitable contributions in respect of excess profits duty, the Executive Committee decided to consider the desirability of applying for a similar concession in respect of income-tax. A Sub-Committee has been appointed, consisting of Sir Walter Trower, Sir Basil Mayhew, Mr. Griffiths, and Mr. Hopkinson, to consider the question. Their inquiry, the result of which will now form part of the Fund's evidence for Lord Cave's Committee, had not been completed by the end of the year.

#### Personnel.

The report states that the Fund suffered a severe loss by the sudden death of Lord Bessborough, the Chairman of the Executive Committee, who had done conspicuously able service to the Fund for many years. It records the death of Sir Henry Burdett, who had been conspicuously active in the service of the Fund in its foundation and during his life, and of two other members of the Council, Lord Murray of Elibank and Sir T. Vezey Strong; also of three visitors, Mr. Heathcote Long, Mr. Howard Morley, and Lord Glenconner; and the retirement from the chairmanship of the Distribution Committee of Sir John Tweedy, one of their oldest and most capable members. The Council express their most grateful thanks to Mr. John G. Griffiths, who has resigned the position of Hon. Secretary, after giving invaluable services to the Fund in this capacity for nine years. It regrets the retirement of three of its members: the Marquis of Cambridge, to whom the Fund is greatly indebted for his services as one of the three Governors of the Fund during the minority of the Prince of Wales, and who resigned his seat on leaving London, and the Rev. W. L. Watkinson and Mr. Will Crooks, who have been compelled to retire through ill-health.

Special Needs.

The report concludes with the following paragraph:

All who believe in the voluntary system must make a special effort to support the hospitals during the present crisis, and to ensure that voluntary contributions form at least a substantial part of their income in the future. The Fund has helped to meet the first need by the emergency distribution of £250,000. If liberally supported by the public it can help greatly with the second, especially in the case of hospitals which are most in need. The Council are greatly indebted to the newspaper press for the assistance they have rendered in the past, and continue to rely upon their kindly co-operation in obtaining for the Fund wider publicity and support.

Lady Hall having seconded the resolution, the adoption of the report was then put and carried unanimously.

On the motion of Lord Stuart of Wortley, seconded by the Rev. Dr. W. T. A. Barber, a resolution was passed delegating further powers to the King's Fund Policy Committee.

### H.R.H. the President's Speech.

The Prince of Wales, who was warmly received, read the following message from H.M. the King:—

"The King is glad to have the opportunity of to-morrow's meeting of King Edward's Hospital Fund to say how, in following its proceedings, he realises what exceptionally heavy demands have lately been made upon the Executive, Distribution and Policy Committees. His Majesty is sure that all well-wishers of the Fund share his feelings of gratitude to the members of these Committees for the time and labour which they have given in dealing with the many important questions submitted to them during the past few months.—Stamfordham."

His Royal Highness said the past was an extremely busy year, with three distributions instead of one, and discussions about the financial difficulties of the hospitals. The way in which the financial difficulties of the hospitals are to be solved was in the very capable hands of Lord Cave's Committee, and the business of the Council is to assist that Committee in every way possible. That is being done through the King's Fund Policy Committee. The Fund were very grateful indeed to Sir Arthur Stanley and the Joint Committee of the British Red Cross Society and the Order of St. John of Jerusalem for asking this Fund to assist them in the splendid work they are doing in connection with the surplus Red Cross funds for the benefit of disabled and all ex-Service men. They contributed the funds, the Council contributed the knowledge and experience of the London hospitals possessed particularly by its Distribution Committee. They were all very glad that Lord Cave's Committee had reported so decidedly in favour of the voluntary system of hospital management. It was for the maintenance of that system that King Edward founded the Fund, and that it still exists. He thought everybody there was convinced that it is the best system both for the patients and for the public, and is glad that the evidence has already brought Lord Cave's Committee unanimously to the same opinion. He expressed on his own behalf as President, and on behalf of the Council, most sincere thanks for the splendid way the Executive and Distribution Committees have given their services. More especially thanks were due to Lord Stuart of Wortley and to the Hon. Secretaries and their staff.

Lord Donoughmore, in proposing a vote of thanks to the Prince of Wales, said they all appreciated the amount of work His Royal Highness had done and the efforts he made to keep in touch with the hospitals.

The Marquess of Crewe, in seconding the resolution, said these were anxious days for everybody, and anxious days also for King Edward's Hospital Fund, and it added great strength and comfort to all connected with the Fund to know that His Royal Highness was President, and took the interest in it which he had shown. He felt also that, among the countless activities of His Royal Highness in all the fields of national life, there could be none which would appeal to him more closely than this work in which His Majesty preceded him, and with which was also connected the name of his illustrious grandfather.

The Prince of Wales, in acknowledging the vote of thanks, said it was very kind of Lord Donoughmore to say what he had said, because he did try to keep in touch with the hospitals, and whenever he got the opportunity he went round to see as many of them as he could.

